# Text4Hope Effectiveness in Reducing Psychological Symptoms among Young Adults in Canada: Longitudinal and Naturalistic Controlled Program Evaluation

**DOI:** 10.3390/jcm12051942

**Published:** 2023-03-01

**Authors:** Belinda Agyapong, Reham Shalaby, Wesley Vuong, April Gusnowski, Shireen Surood, Andrew J. Greenshaw, Yifeng Wei, Vincent Israel Opoku Agyapong

**Affiliations:** 1 Department of Psychiatry, Faculty of Medicine and Dentistry, University of Alberta, Edmonton, AB T6G 2B7, Canada; 2 Addiction and Mental Health, Alberta Health Services, Edmonton, AB T2S 3C3, Canada; 3 Department of Psychiatry, Faculty of Medicine, Dalhousie University, Halifax, NS B3H 2E2, Canada

**Keywords:** young adults, mental health, naturalistic controlled trial, anxiety, thoughts of self-harm, death wish, Text4Hope, stress, depression

## Abstract

Background: Chronic stress, anxiety, and depression are psychological problems that can hurt young adults, interfering with their everyday function, academic achievement, and interpersonal relationships. This study aimed to assess the impact of Text4Hope, an online mental health service, on the psychological well-being of young adults. Methods: This study adopted both longitudinal and naturalistic controlled trial designs. It examined clinical outcomes in young adult (≤26 years old) subscribers of Text4Hope who completed surveys at baseline and six weeks and compared clinical parameters in two groups of subscribers. The first group comprised the intervention group (IG) (young adult subscribers who received once-daily supportive text messages for six weeks and completed sixth-week evaluation measures between 26 April and 12 July 2020), and the second group was the control group (CG) (young adult subscribers who joined Text4Hope in the same time frame and completed a baseline survey and were yet to receive any text messages). The prevalence of moderate to high stress, anxiety, and depression was measured at baseline and six weeks in the longitudinal study and between the two groups for the naturalistic controlled study using the Perceived Stress Scale (PSS-10), Generalized Anxiety Disorder 7-item (GAD-7), and Patient Health Questionnaire-9 (PHQ-9). Inferential statistics, including the *t*-test, McNemar test, chi-square, and binary logistic regression analyses, were used to evaluate the differences in the prevalence and severity of the psychological symptoms. Results: In the longitudinal study, of the 9214 subscribers to Text4Hope who completed the baseline survey, 1047 (11.4%) were identified as youth. For the young adult subscribers who completed both the baseline and sixth-week surveys (n = 114), a significant reduction in the prevalence of moderate to high stress (8%) and likely GAD (20%) from baseline to six weeks was reported. Similarly, there was a significant reduction in the mean scores on the PSS-10, GAD-7, and Composite Mental Health score but not the PHQ-9 from baseline to six weeks. The largest reduction in mean scores was for the GAD-7 scale (18.4%), with a small effect size overall. For the naturalistic study, the IG included 173 young adult subscribers of Text4Hope who completed the sixth-week survey compared to 92 subscribers in the CG who completed the baseline survey during the designated period. There was a significantly lower prevalence for likely Moderate Depressive Disorder (MDD) (25.2%) and suicidal thoughts/thoughts of self-harm (48.4%), with a small effect size in the IG compared to the CG. Similarly, lower mean scores were reported for all outcome variables in the IG compared to the CG, with a small to medium effect size. The receipt of daily supportive text messages for six weeks resulted in significantly lower odds of both likely GAD and experiencing thoughts of self-harm or death wish while controlling for sociodemographic characteristics. Conclusions: The Text4Hope service is an effective tool for mental health support for young adult subscribers. Young adults receiving the service exhibited a reduction in psychological symptoms, including thoughts of self-harm or death wish. This population-level intervention program can be used to effectively support young adult mental health and in suicide prevention programs.

## 1. Introduction

Young adults experience many emotional or behavioural difficulties, increasing their vulnerability to mental health and psychological issues [1]. Stress occurs in response to an external demand perceived by an individual that exceeds their capability to handle the challenge imposed by that demand [2]. Often for young adults, the personal evaluation of the nature of the demand, their skills, the resources available, and the presumed outcomes will determine the quality and significance of the stress experience [2]. For instance, chronic stress may lead to increased alcohol and drug consumption, resulting in inappropriate anger [3,4]. Chronic stress is also a risk factor for developing mental distress or psychiatric illnesses, such as anxiety and depression, and can affect young adults’ everyday function and emotional balance [5,6,7]. Depression and anxiety are common disorders among young adults. Published results indicate that individuals with depression often have more significant psychological problems and may experience difficulties in meeting interpersonal tasks and time-management, physical, and productivity demands; decreased work quality; and increased absences due to the fact of illness, all of which can have profound effects on productivity [8,9,10]. Anxiety and mood disorders such as depression are comorbid, prevalent, distressing, and cause impairment [11,12,13]. These disorders negatively impact young adults, interfering with academic achievement and interpersonal relationships, and comprise a risk factor for suicide and other psychopathology [14,15]. According to Kendall, anxiety disorders are highly comorbid and often present as multiple disorders with overlapping symptoms. This study also reported that 55.3% of the respondents met the criteria for at least one DSM-IV disorder which was not the primary target [16]. Other literature reports a variable prevalence of anxiety and depression among young adults [17,18,19].

The onset of the COVID-19 pandemic led governments worldwide to introduce lockdowns to curtail the spread of the infection. After almost three years, the threat of COVID-19 variants remains a concern due to mutations that seem to increase infection rates [20]. In general, the most frequent sources of perceived stress were the uncertainty about the pandemic, its duration, and the disruption of social life and other essential activities. This led to a surge in mental health problems, especially among young adults [21]. One study [22] determined the prevalence of GAD and depressive symptoms in the Chinese general public to be 35.1% and 20.1%, respectively. Both GAD and depressive symptoms were significantly higher in those younger than 35 years compared to participants aged 35 years or older [22]. In Switzerland, approximately 20% of young adults met the criteria for at least one mental health issue, such as depression or GAD, and one-third of children/adolescents screened positive for at least one of the mental health problems, such as depression or anxiety [21]. Nwachukwu et al., in Canada, also found that the mean scores on the Perceived Stress Scale (PSS), Generalized Anxiety Disorder 7-item scale (GAD-7), and Patient Health Questionnaire-9 (PHQ-9) scale were high for individuals aged 25 years or younger (i.e., young adults) [23]. The study suggested that young adults are more prone to and disproportionally affected by mental health issues. The management of these psychological issues requires innovative, cost-effective, easily scalable population-level interventions [24,25]. Many interventions have been geared towards preventing the deterioration or possibly onset of these mental disorders among young people, and the use of technology is generally seen as natural and desirable among young adults [1]. Globally, mobile phone ownership and access are high, and advances in mobile technology are shaping digital communications [26].

Furthermore, mobile phones are widely used by young adults, who generally utilize e-communication technologies, such as instant messaging, far more than older adults [27]. Increasing evidence indicates that SMS text messaging may be the preferred way to deliver adolescent health services that promote psychological well-being and reduce the risk factors to adolescent mental health [28]. Hence, cognitive behavioural therapy-based supportive text messages can be easily accepted among young adults and adopted as an intervention to support their mental health. Cognitive-behavioural-based supportive mobile text messaging programs constitute a unique and innovative approach, providing a low-cost, convenient, and easily scalable way of delivering psychological interventions to individuals with mental health problems [29], and they have been used effectively for the general population in Alberta to support their mental health [24,25]. Supportive text messaging may effectively reduce depression and increase abstinence duration in alcohol use disorder [30,31]. This study examined the impacts of a supportive text messaging program, Text4Hope, on the severity and prevalence of stress, anxiety, and depression among young adults during the COVID-19 pandemic.

## 2. Methodology

### 2.1. Study Setting and Design

The study was conducted in Alberta, a province in western Canada, with an estimated population of 4,601,314 in October 2021 [32], including 545,900, or 11.9%, youth. Text4Hope is a remotely delivered service stimulated by the pandemic to integrate technology-based mental health support provided to the general population in Alberta during the COVID-19 pandemic [33]. This study was, however, designed to focus on the assessment of the Text4Hope outcomes among only the young subscribers of the Text4Hope program.

This study employed two designs, including a longitudinal design and a naturalistic controlled trial design, focusing on young adults (<26 years) subscribed to the Text4Hope service. The longitudinal study examined clinical outcomes among the Text4Hope subscribers who completed surveys at baseline and again at six weeks. The naturalistic controlled study compared two study populations of the Text4Hope young adult subscribers; the first group was an intervention group (IG) (i.e., Text4Hope subscribers who received a once-daily supportive text messages for six weeks and completed sixth-week evaluation measures between 26 April and 12 July 2020), and the second group served as a control group (CG) (young adults who joined Text4Hope in the same time frame and completed baseline surveys and were yet to receive any text messages).

### 2.2. Ethics Statement

The study was conducted according to the guidelines of the Declaration of Helsinki and ethical principles for medical research involving human subjects. The study protocol [33] was approved by the University of Alberta Reviews and Ethics Board (Pro00086163) on 18 March 2020. Informed consent was implied when subscribers completed the online survey and submitted responses, consistent with the University of Alberta Health Research Ethics Board approval.

### 2.3. Text4Hope Program

Text4Hope is a self-subscription English program [24] whereby subscribers receive daily supportive SMS text messages for three months by texting the word “COVID19HOPE” to a short code number. The messages were written within a cognitive behavioural framework by mental health professionals. The first message welcomes subscribers to the service and allows them to complete a baseline survey. For the IG, after six weeks, they had the opportunity to receive another text message link inviting them to complete a follow-up survey, while the CG were yet to receive this follow-up survey message. Previous publications provide detailed comprehensive descriptions of the protocol for this intervention [25,34].

### 2.4. Data Collection

Participation in the Text4Hope service and receiving supportive SMS text messages were independent of the voluntary completion of the online surveys. Subscribers were able to opt-out of the service at any time. Daily supportive text messages were delivered by a computer program at 9:00 a.m. Mountain Time each day. The surveys captured various sociodemographic data provided as categorical factors. For analysis, the variable categories were further collapsed, including sex at birth (male, female, and other), educational level (postsecondary education and high school diploma or less), age (≤25 yrs), ethnicity (White, Indigenous, Asian, and other), employment status (employment, not employed, student, and retired), relationship status (married, cohabiting or partnered, separated or divorced, single, and other), and housing status (own home, live with family/friend, renting, and other). The study outcome measures focused on the differences in the severity and prevalence of stress, anxiety, and depression symptoms, using English versions of validated self-reported screening scales, including the Perceived Stress Scale (PSS; PSS score ≥ 14 indicates moderate or high stress) [35], the Generalized Anxiety Disorder 7-item (GAD-7) scale (GAD-7 score ≥ 10 indicates likely generalized anxiety disorder (GAD)) [36], and the Patient Health Questionnaire-9 (PHQ-9; a score ≥ 10 indicates likely major depressive disorder (MDD)) [37]. The PSS is a 10-item questionnaire with a Cronbach’s alpha of >0.70 and is used to assess the level of stress in the previous month [35]. The GAD-7 is a 7-item questionnaire with a Cronbach’s alpha of 0.92 and is used to assess the self-reported levels of anxiety in respondents in the two weeks prior to assessment [36]. The PHQ-9 is a 9-item questionnaire with a Cronbach’s alpha of 0.89 and is used to assess the severity of depression symptoms [37].

The data for the longitudinal trial were collected between 20 March 2020, and 12 July 2020, and the data for the naturalistic controlled trial were collected between 26 April and 12 July 2020. The clinical assessment questionnaires were the same for all subscribers at baseline and six weeks. Each survey took ten minutes to complete, and no incentives were used.

### 2.5. Outcome Measures

The primary outcome measures of interest for this study were the differences in the prevalence of clinically meaningful (moderate to high) stress, anxiety, depression symptoms, and thoughts of self-harm or death wish measured using the ninth question on the PHQ-9 from baseline to six weeks for the longitudinal study and between the IG (six weeks prevalence) and the CG (baseline prevalence) for the naturalistic trial.

The secondary outcome measures included the differences in the mean scores on the PSS-10, GAD-7, and PHQ-9 and the Composite Mental Health (CMH) score, which was defined as the sum of the scores on the three scales between the baseline and six weeks scored participants in the longitudinal study and between the IG at six weeks and the CG at baseline in the naturalistic trial. For all study participants in the naturalistic controlled trial (controlling for demographic variables), another secondary outcome variable of interest was to determine whether, when controlling for demographic variables, the daily supportive text messages contributed to lower odds of moderate to high stress, GAD symptoms, and MDD symptoms in young adults.

### 2.6. Hypothesis

We hypothesized that the prevalence of moderate/high stress, likely GAD, likely MDD, and thoughts of self-harm/death wish would be 20% lower at six weeks compared to the baseline for the group who completed the surveys at the two time points for the longitudinal study and 20% lower in the IG compared with the CG in the naturalistic controlled trial. We also hypothesized that being in the IG would independently predict the absence of moderate to high stress, likely GAD, and likely MDD symptoms when all demographic variables were controlled for in a logistic regression model. In addition, we hypothesized that for young adult subscribers to Text4Hope who completed surveys at the two time points, there would be at least 20% lower mean scores on the PSS-10, GAD-7, and PHQ-9 and the mean CMH score at six weeks compared with their baseline scores. Finally, we hypothesized that young adult subscribers to Text4Hope in the IG would have at least 20% lower mean scores on the PSS-10, GAD-7, and PHQ-9 and the mean CMH score compared with the corresponding mean scores for the CG participants. These hypotheses were based on the more than 20% decrease in depression symptom scores in the treatment group compared to the control group observed in two RCTs in Ireland and Canada [38,39], as well as the greater than 20% reduction in anxiety symptom scores from baseline to six weeks and three months in the general population of subscribers to Text4Hope [24,25].

### 2.7. Sample Size Considerations

For the longitudinal study, we determined, using an online script [40], that a sample size of 82 would be sufficient to detect differences in the baseline and sixth-week prevalence for moderate to high stress, with an 80% power, assuming a 90% prevalence of moderate to moderate to high stress at baseline, a hypothesized difference of 20% between the baseline and sixth-week prevalence, a two-sided significance of 5% for detecting a difference, a correlation between a paired observation of 0%, and after applying continuity correction.

For the naturalistic controlled trial, to detect a 20% difference in the prevalence of moderate to high stress between the IG and the CG, based on a power of 80% (β = 0.2) and a two-sided significance level of α = 0.05, and assuming a 90% prevalence of moderate to high stress at baseline for the CG, we estimated, using an online script [41], that a sample size of 73 per group would be sufficient.

### 2.8. Statistical Analysis

The data were analyzed using SPSS for Windows, version 25 (IBM Corporation, New York, USA) [42]. For the longitudinal study, sociodemographic and clinical variables were examined between the participants who completed both the baseline and sixth-week surveys and participants who completed only the baseline survey, employing the chi-square or Fisher’s exact test. We used a paired sample *t*-test and the McNemar test, respectively, to assess the differences in the mean scores of the PSS-10, GAD-7, and PHQ-9 and the CMH scores, as well as the prevalence of moderate to high stress, likely GAD, likely MDD, and thoughts of self-harm/death wish from baseline to six weeks for young adult subscribers who completed surveys at the two-time points.

For the naturalistic controlled trial, demographic characteristics were examined between the two study groups, IG and CG. Furthermore, we examined the prevalence of clinically meaningful psychological problems between the IG and CG at baseline to ascertain if there were differences using the chi-square test.

The differences in the mean scores of the PSS-10, GAD-7, and PHQ-9 and the CMH scores between the IC and CG were examined using independent sample *t*-tests. Similarly, we examined the prevalence of moderate to high stress, likely GAD, likely MDD, and thoughts of self-harm/death wish in both the IG and CG using the chi-square/Fisher’s exact tests. The results are summarized by numbers and percentages and compared by chi-square analysis with a two-tailed criterion (α < 0.05), using the same score for the PSS-10, GAD-7, and PHQ-9.

To assess the independent impact of the Text4Hope daily supportive messages on the presence of moderate to high stress, likely GAD, and likely MDD on young adult subscribers at six weeks, all demographic predictors and “IG” variables were imputed into three different binary logistic regression models. The odds ratios from the regression models were examined to determine the associations between “receiving the daily supportive messages” and the likelihood of participants self-reporting symptoms consistent with moderate to high stress, likely GAD, likely MDD, and thoughts of self-harm/death wish while controlling for sociodemographic variables (i.e., gender, ethnicity, education level, employment status, relationship status, and housing status) in the respective models. Correlation analyses were performed before the regression analysis to rule out strong correlations (r_s_ ≥0.7) among the predictor variables. There was no missing data imputation, and the total numbers reported represent the total responses recorded for each variable.

## 3. Results

**A**  **Longitudinal study outcomes**

**Figure 1:**
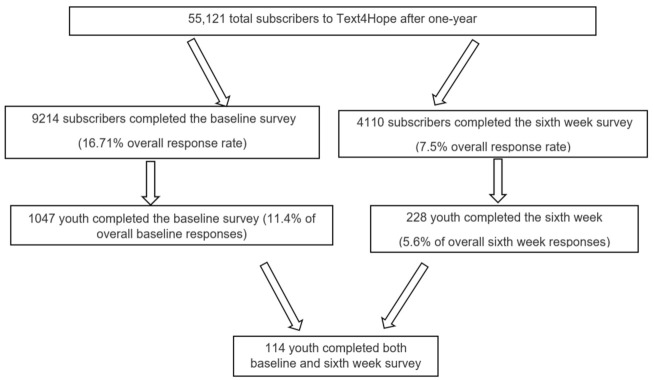
Study flow chart.

Figure 1 shows that the response rates for the baseline and sixth-week surveys were 16.71% and 7.5%, respectively. Of the 9214 subscribers to Text4Hope who completed the baseline survey, 1047 identified as youth (≤25 years of age), representing 11.4%. Only 114 youths completed both the baseline and sixth-week surveys and were included in the longitudinal outcome analysis.

Table 1 shows the distribution and comparison of the demographic and clinical characteristics of the participants who completed both the baseline and sixth-week surveys and the participants who completed only the baseline survey.

Table 1 suggests that participants who completed both the baseline and sixth-week survey were similar in their demographic characteristics, except in their gender for which a higher proportion of participants in the former group self-identified as other gender compared to the latter group. Similarly, respondents in the two groups were similar in their clinical characteristics, except that the mean score for the GAD-7 scale was higher in the participants in the longitudinal study.

For young adult subscribers who completed both the baseline and six weeks surveys, changes in the prevalence of moderate to high stress, likely GAD, likely MDD, and thoughts of self-harm/death wish from baseline to six weeks were assessed using the McNemar Test, as shown in Table 2.

Table 2 suggests statistically significant reductions in the prevalence of moderate to high stress and likely GAD but not likely MDD and thoughts of self-harm/death wish from baseline to six weeks for young adult subscribers who completed both the baseline and sixth-week surveys. The largest reduction in prevalence was for likely GAD (20%).

We ran a paired sample *t*-test to assess the changes in mean scores on the PSS, PHQ-9, and GAD-7 scales and the CMH score, as shown in Table 3.

Table 3 suggests a statistically significant reduction in mean scores on the PSS-10, GAD-7, and CMH but not the PHQ-9 from baseline to six weeks. The largest reduction in mean scores was for the GAD-7 scale (18.4%), with a small effect size overall.

**B**  **Naturalistic controlled trial**

The total number of young adults who completed the Text4Hope survey either at baseline or at six weeks was 265, with 173 (65.3%) in the IG and 92 (34.7%) in the CG. Table 4 illustrates the demographic characteristics of respondents in the IG and CG.

Table 4 suggest that the IG and CG were similar in their demographic characteristics, except for their employment status, where the IG had 16.8% of participants reporting they were unemployed compared with 0% in the CG.

### 3.1. Differences in the Prevalence of Psychological Problems between the IG and CG

We used a chi-square test to ascertain if there were differences in the baseline prevalence of clinically meaningful psychological problems between the IG and CG, as shown in Table 5.

The results in Table 5 suggest that before either group received any supportive text messages, the prevalence of moderate to high stress, likely GAD, likely MDD, and thoughts of self-harm/death wish were similar for the IG and the CG.

We assessed the impact of the Text4Hope program on young adult subscribers by using the chi-square test to compare the IG sixth-week and the CG baseline prevalence of moderate to high stress, likely GAD, likely MDD, and thoughts of self-harm or death wish measured within the same time frame for the two groups, as shown in Table 6.

Table 6 shows a significantly lower prevalence in the IG compared to the CG for likely MDD and suicidal thoughts/thoughts of self-harm, with a small effect size. The difference in the prevalence between the IG and CG was 25.2% for likely MDD and 48.4% for thoughts of self-harm/death wish. There was no statistically significant difference between the IG and CG concerning the prevalence of moderate to high stress and likely GAD.

To assess the impact of the sociodemographic variables on the likelihood for young adult subscribers in the IG to present with moderate to high stress, likely GAD, likely MDD, and thoughts of self-harm/death wish, we entered the treatment group (IG vs. CG) as a variable together with all sociodemographic variables into four separate logistic regression models. The key findings from the four models are summarized in Table 7.

Table 7 shows the key results from four separate logistic regression models, which assessed the independent contribution of the treatment group variable to the presence of moderate to high stress, likely GAD, likely MDD, and thoughts of self-harm or death wish among all study participants while controlling for sociodemographic variables.

The results in Table 7 demonstrate that the treatment group variable was a significant predictor of likely GAD and thoughts of self-harm or death wish (*p* ≤ 0.05) but not the moderate to high stress and likely MDD (*p* ≥ 0.05) among the study participants, while controlling for sociodemographic variables (including ethnicity, educational status, and relationship status, which had no significant differences between the IG and CG on chi-square analysis, as well as employment status for which there was a significant difference between the IG and CG, as shown in Table 4) in the two models.

In terms of likely GAD, the model was not significant, *x*^2^ (df = 18, *n* = 189) = 26.15 *p* > 0.05, explaining between 13% (Cox and Snell R2) and 18% (Nagelkerke R2) of the variance and correctly classifying 68% of all cases. However, controlling for all demographic characteristics, the “treatment group” contributed significantly to the model (Wald = 4.8). The IG was 0.44 times less likely to present with likely GAD during the study period than CG (OR = 0.44; 95% CI = 0.21–0.92). This suggests that participants in the CG were 2.27 times more likely to meet the cut-off threshold for likely GAD than participants in the IG, controlling for other variables in the regression model.

For experienced thoughts of self-harm or death wish, the full model was significant, *x*^2^ (df = 18 *n* = 194) = 33.76, *p* = 0.01, explaining between 16% (Cox and Snell R2) and 21% (Nagelkerke R2) of the variance and correctly classifying 66% of all cases. Controlling for all demographic characteristics, the “intervention arm” contributed significantly to the model (Wald = 6.52). The IG was 0.42 times less likely to have thoughts of self-harm or death wish during the study period than CG (OR = 0.42; 95% CI = 0.21–0.82). Invariably this implies that participants in the CG were 2.38 times more likely to have thoughts of self-harm or death wish than participants in the IG, controlling for other variables in the regression model.

### 3.2. Differences in Mean Scores on Psychological Symptom Rating Scales between Intervention and Control Groups

Table 8 shows the results of an independent sample *t*-test assessing differences in mean scores between the IG and CG for the PSS-10, GAD-7, PHQ-9 and CMH scores.

From Table 8, there was a significantly lower mean score for the IG at six weeks compared to the CG at baseline across all outcome variables (*p* < 0.05). The mean scores on the PSS-10, PHQ-9, and GAD-7 and the CMH score were significantly higher for the CG at baseline compared to the IG at six weeks by 8.6%, 25.7%, 23.6%, and 18.7%, respectively.

## 4. Discussion

Text4Hope is a text-based program that provides mental health support to the public, including young adults, during the COVID-19 pandemic. These longitudinal and naturalistic controlled studies compared the clinical outcomes for young adult subscribers of Text4Hope. For the longitudinal study, young adult Text4Hope subscribers who completed surveys at baseline and six weeks were included in the study. For the naturalistic controlled trial, the IG young adult subscribers received daily supportive text messages for six weeks and completed the sixth-week follow-up survey, while the CG young adult subscribers signed up for the Text4Hope program during the same time frame and completed the baseline survey and had not yet received any text messages. The key findings from the longitudinal study were that there were statistically significant reductions in the prevalence of moderate to high stress and likely GAD from baseline to six weeks by 7.56% and 20.33%, respectively, but not likely MDD and thoughts of self-harm or death wish for young adult subscribers who completed surveys at both time points. In addition, the mean scores on both the PSS-10 and GAD-7 were significantly lower at six weeks compared to the baseline for subscribers who completed surveys at both time points (4.31% and 18.4% reduction in mean scores, respectively), indicating clinical improvement in the subscribers. The key findings from the naturalistic controlled study were that significant differences were observed in the prevalence of likely MDD and thoughts of self-harm or death wish but not moderate to high stress and likely GAD. However, the treatment group was a significant independent predictor of likely GAD and thoughts of self-harm or death wish but not moderate to high stress and likely MDD when demographic variables were controlled for in separate regression models. The participants in the IG had significantly lower odds of presenting with both likely GAD and thoughts of self-harm or death wish in the regression models. Finally, the mean scores on the PSS-10, PHQ-9, and GAD-7 and the CMH score were significantly higher for the CG compared to the IG by 8.6%, 25.7%, 23.6%, and 18.7%, respectively, indicating more severe mental health problems in the CG. Thus, the result of the study appears to extend the evidence indicating the effectiveness of Text4Hope in reducing the severity of stress, anxiety, and depression, as well as the prevalence of thoughts of self-harm or death wish and likely MDD, among young adults.

Finding cost-effective and easily scalable population-level interventions [43] to address anxiety symptoms among young adults is a priority for health policymakers and governments. Young adults are generally prone to high levels of anxiety, which are usually comorbid with other mental health problems [16]. The mean scores for anxiety in the Text4Hope subscribers were reported as highest among young adults during the pandemic [23]. This study indicates that daily supportive text messages delivered to young adults may reduce the severity and prevalence of psychological symptoms, particularly moderate to high anxiety symptoms, in young adults by approximately 20% in both the longitudinal study and in the naturalistic controlled study, which is partly consistent with both our study hypothesis and with the results reported for the general population of subscribers to Text4Hope [24,25]. The regression analysis results, which controlled for the demographic characteristics of the respondents, suggest that daily supportive text messages effectively mitigate anxiety symptoms among young adults irrespective of the current sociodemographic characteristics. The results of this study extend the literature with evidence of the benefits of Text4Hope, specifically for young adults.

Concerning the severity and prevalence of depression, which are frequently comorbid with anxiety [16,44], the nonsignificant 9.3% difference between the baseline and six weeks prevalence observed in the longitudinal study and the significant 25% difference in the severity of symptoms between the IG and the CG recorded in the naturalistic controlled trial are only partly consistent with our stated hypothesis. The results of the naturalistic study, however, confirm previous studies by Agyapong et al., which demonstrated the effectiveness of daily supportive text message interventions for reducing depression symptoms through several controlled clinical trials [30,31,38,45], as well as evaluations of population-level initiatives [24,25,46,47] delivered as part of the ResilienceNHope suite of programs [48,49]. This notwithstanding, when sociodemographic factors were controlled for in the regression model, the intervention group was not significantly associated with lower odds of presenting with likely MDD symptoms in young adult subscribers to Text4Hope, unlike GAD symptoms.

The results also suggest that the Text4Hope program had the least impact on the stress levels of young adult subscribers. For the longitudinal study, there was a significant reduction in the prevalence of moderate to high stress by only 7.56% and a reduction in the mean score on the PSS-10 of only 4.3% from baseline to six weeks. Similarly, for the naturalistic trial, there was only an 8.6% difference between the IG and the CG in terms of the mean scores on the PSS-10. Although this was statistically significant, the difference in the mean scores was much lower than that for our stated hypothesis. The lower mean difference in mean scores for stress, compared to anxiety and depression, is consistent with what was reported for general population subscribers to Text4Hope [24,25]. Similar to what has been reported for MDD, when sociodemographic factors were controlled for in the regression model, the intervention group was not significantly associated with lower odds of presenting with moderate to high stress symptoms.

Among young adults, the lack of apparent effectiveness of the daily supportive text message intervention to significantly reduce symptoms of both stress and depression when controlling for the included sociodemographic characteristics has never been previously examined or reported. Thus, this study extends the literature on this important topic. The study results also indicated significantly lower odds for IG subscribers of Text4Hope to have thoughts of self-harm or death wish than the CG subscribers. A survey by Randall et al. conducted among young adults reported a high prevalence of suicide attempts, with 23.2% of the respondents reporting they had thought about suicide, and 28.3% reporting they had made a suicide attempt [50]. In Canada, suicide remains the second most important cause of death of persons between 15 and 34 years of age, including young adults [51]. This suggests that young adults are at a higher risk of experiencing thoughts of self-harm or death wish. Our study results indicate that CBT-based text messages may help to reduce this phenomenon among young adults. Young adults outnumber adults in using text messages [27]. Similarly, texting was the preferred mode of communication among adolescents with their physicians, compared to face-to-face interactions [28]. Although these interventions aimed at reducing psychological symptoms, they may have inadvertently improved the youth’s academic achievement and interpersonal relationships [14,15]. This may also invariably lead to a reduction in other comorbid mental health illnesses, since mental health problems are usually concurrent and comorbid among young adults [16]. Hence, the text message intervention can be appropriately and successfully used to reduce symptoms of stress, anxiety, depression, and thoughts of self-harm or death wish among youth.

### Limitations of the Study

This study is not without limitations. First, for the both the longitudinal and naturalistic studies, the sample size and prevalence for likely MDD and likely GAD at baseline were smaller than the projected sample size and baseline prevalence for the two conditions. Thus, both studies might have been underpowered to detect differences in some of the included variables. Second, the low response rate at both baseline and six weeks means that the prevalence for the psychological conditions reported at both time points may be different for the majority of Text4Hope youth subscribers who might not have completed the surveys. Thus, our study findings may not be generalizable to all Text4Hope youth subscribers. Third, the included naturalistic study design does not follow the conventional randomization of the participants into the two study groups at baseline. However, as reported in this paper (Table 5), before either group received any supportive text messages, the prevalence of moderate to high stress, likely GAD, likely MDD, and thoughts of self-harm/death wish were similar for the IG and the CG. In addition, the impact of the potential differences in clinical variables based on differences in sociodemographic factors was controlled for using binary logistic regression analysis. Third, although validated and standardized proxy scales, the instruments used to assess mental health outcomes are simply not diagnostic. Despite these limitations, this study provides important insights into and evidence on the effectiveness of Text4Hope in helping to alleviate the mental health burden for young adults.

## 5. Conclusions

This study demonstrates that independent of sociodemographic variables, the prevalence of likely GAD symptoms and thoughts of self-harm or death wish may be significantly lower in young adults who were enrolled for six weeks to receive daily supportive text messages (Text4Hope program). This is particularly encouraging, as young adults have already adapted to SMS text messaging and texting. Therefore, this mode of intervention can be used to supplement existing treatments for psychological problems impacting young adults. In addition, the cost effectiveness and easy scalability of supportive text message interventions mean that policymakers and governments can quickly implement similar programs as part of national youth suicide prevention strategies.

## Figures and Tables

**Table 1:** Distribution of the demographic and clinical characteristics between the participants who completed both the baseline and sixth-week survey and the participants who completed only the baseline survey.

Variable	Participants Completed Both Baseline and Sixth-Week Surveys, *n* (%) N = 114	Participants Completed Only Baseline Survey, *n* (%) N = 932 ^a^	Total	Chi^2^/*t*-Test Value (df) ^b^	*p*-Value
Gender					
Male	13 (11.4)	121 (13.0)	134 (12.8)	6.54	0.04
Female	94 (82.5)	790 (84.9)	884 (84.6)		
Other	7 (6.1)	20 (2.1)	27 (2.6)		
Ethnicity					
White	88 (77.2)	654 (70.4)	742 (71.1)	4.09	0.25
Indigenous	4 (3.5)	51 (5.5)	55 (5.3)		
Asian	6 (5.3)	96 (10.3)	102 (9.8)		
Other	16 (14.0)	128 (13.8)	144 (13.8)		
Education Level					
Less than high school diploma	25 (21.9)	214 (23.0)	239 (22.9)	0.08	0.99
High school diploma	21 (18.4)	172 (18.5)	193 (18.5)		
Postsecondary education	67 (58.8)	536 (57.6)	603 (57.7)		
Other education	1 (0.9)	9 (1.0)	10 (1.0)		
Employment Status					
Employed	39 (34.2)	356 (38.2)	395 (37.8)	6.85	0.08
Unemployed	19 (16.7)	203 (21.8)	222 (21.2)		
student	54 (47.4)	334 (35.8)	388 (37.1)		
Other	2 (1.8)	39 (4.2)	41 (3.9)		
Relationship Status					
Married, cohabiting, or partnered	52 (45.6)	465 (50.1)	517 (49.6)	2.42	0.49
Separated or divorced	2 (1.8)	7 (0.8)	9 (0.9)		
Single	59 (51.8)	441 (47.5)	500 (47.9)		
Other	1 (0.9)	16 (1.7)	17 (1.6)		
Housing Status					
Owns a home	6 (5.3)	86 (9.4)	92 (8.9)	2.37	0.5
Living with family/friend	67 (58.8)	532 (58.1)	599 (58.2)		
Renting	39 (34.2)	286 (31.2)	325 (31.6)		
Other	2 (1.8)	12 (1.3)	14 (1.4)		
Likely Stress					
At most mild stress	2 (1.8)	32 (3.7)	34 (3.5)	0.97	0.32
Moderate to high stress	107 (98.2)	841 (96.3)	948 (96.5)		
Likely MDD					
At most mild depression	30 (28.8)	262 (31.9)	292 (31.6)	0.4	0.53
Moderate to severe depression	74 (71.2)	559 (68.1)	633 (68.4)		
Likely GAD					
At most mild GAD	25 (24.5)	274 (34.0)	299 (33.0)	3.72	0.054
Moderate to severe GAD	77 (75.5)	531 (66.0)	608 (67.0)		
Total Score of PSS-10					
Mean score (SD)	N = 109	N = 873	-	1.90 (148.43) ^c^	0.06
	26.62 (5.41)	25.56 (6.40)			
Total Score of PHQ-9					
Mean score (SD)	N = 104	N = 821	-	0.86	0.39
	14.01 (6.52)	13.40 (6.81)		−923	
Total Score of GAD-7					
Mean score (SD)	N = 102	N = 805	-	2.41	0.02
	13.73 (5.14)	12.29 (5.73)		−905	

^a^ Total is variable according to the completed responses provided; ^b^ df: degree of freedom; ^c^ Welch’s *t*-test was used. MDD—Moderate Depressive Disorder, GAD—Generalized Anxiety Disorder, PHQ—Patient Health Questionnaire, PSS—Perceived Stress Scale.

**Table 2:** Changes in the prevalence of moderate to high stress, likely GAD, and likely MDD.

Clinical Condition	Prevalence *n*/N (%)		Change from Baseline, %	(df)	*p*-Value
	Baseline	Six Weeks			
**Moderate to high stress**	93/95 (97.9)	86/95 (90.5)	7.56	1	0.02
**Likely MDD**	54/81 (66.7)	49/81 (60.5)	9.30	1	0.42
**Likely GAD**	59/80 (73.8)	47/80 (58.8)	20.33	1	0.01
**Thoughts of self-harm/death wish**	39/81 (48.1)	33/81 (40.7)	15.38	1	0.24

**Table 3:** Changes in baseline mean scores of PSS, PHQ-9, and GAD-7 after the introduction of Text4Hope.

Measure	Scores			Change from Baseline, %	Mean Difference (95% CI)	*p*-Value	*t*-Value	Effect Size (Cohen’s *d*)
	*n*	Baseline Score, Mean (SD)	Sixth-Week Score, Mean (SD)					
**PSS-10 total score**	95	26.46 (5.54)	25.33 (6.75)	4.31	1.14 (0.03–2.25)	0.05	2.03	0.18
**PHQ-9 total score**	81	13.09 (6.38)	12.48 (6.53)	4.66	0.61 (−0.50–1.71)	0.28	1.09	0.09
**GAD-7 total score**	80	13.19 (4.97)	10.76 (5.62)	18.42	2.43 (1.34–3.51)	<0.01	4.45	0.46
**CMH score**	80	51.93 (14.63)	47.84 (17.24)	7.88	4.09 (1.48–7.03)	<0.01	2.77	0.26

CI: confidence interval; CMH: Composite Mental Health score.

**Table 4:** Distribution of the demographic characteristics of the youth subscribers across the intervention groups.

Variable	Control Group	Intervention Group	Total	Chi^2^/Fisher’s Exact *	*p*-Value
**Gender**					
Male	13 (14.1)	15 (8.7)	28 (10.6)	3.48	0.18
Female	78 (84.8)	151 (87.3)	229 (86.4)		
Other	1 (1.1)	7(4.0)	8 (3.0)		
**Ethnicity**					
White	66 (72.5)	129 (74.6)	195 (73.9)	0.31	0.96
Indigenous	4 (4.4)	6 (3.5)	10 (3.8)		
Asian	12 (13.2)	20 (11.6)	32 (12.1)		
Other	9 (9.9)	18 (10.4)	27 (10.2)		
**Education Level**					
Less than a high school diploma	31 (33.7)	28 (20.4)	59 (25.8)	6.42	0.09
High school diploma	18 (19.6)	24 (17.5)	42 (18.3)		
Postsecondary	42 (45.7)	84 (61.3)	126 (55.0)		
Other	1 (1.1)	1 (0.7)	2 (0.9)		
**Employment Status**					
Employed	43 (47.3)	53 (38.7)	96 (42.1)	17.07	0.001
Unemployed	0 (0.0)	23 (16.8)	23 (10.1)		
Student	46 (50.5)	59 (43.1)	105 (46.1)		
Other	2 (2.2)	2 (1.5)	4 (1.8)		
**Relationship Status**					
Married, cohabiting, or partnered	43 (47.3)	62 (45.3)	105 (46.1)	3.64 *	0.27
Separated or divorced	0 (0.0)	2 (1.5)	2 (0.9)		
Single	46 950.5)	73 (53.3)	119 (52.2)		
Other	2 (2.2)	0 (0.0)	2 (0.9)		
**Housing Status**					
Owns a home	6 (6.6)	7 (5.2)	13 (5.8)	2.83	0.42
Live with family/friend	62 (68.1)	84 (62.2)	146 (64.6)		
Renting	21 (23.1)	43 (31.9)	64 (28.3)		
Other	2 (2.2)	1 (0.7)	3 (1.3)		

* Fisher’s exact test was applied.

**Table 5:** Chi-square association test comparing the baseline prevalence of clinically meaningful problems in the IG and CG.

Clinical Condition	Prevalence *n*/N (%)		χ2 (df)	*p*-Value *
	IG (Baseline)	CG (Baseline)		
Moderate to high stress *	88/90 (97.8%)	79/82 (96.3%)	0.31 (1)	0.67
Likely MDD ***	60/86 (69.8%)	62/82 (75.6%)	0.72 (1)	0.49
Likely GAD **	61/84 (72.6%)	59/80 (73.8%)	0.03 (1)	0.99
Thoughts of self-harm/death wish	44/87 (50.6)	42/73 (57.5)	0.77 (1)	0.43

* Moderate or high stress defined as PSS ≥ 14; ** likely GAD defined as GAD-7 ≥ 10; *** likely MDD defined as PHQ-9 ≥ 10. IG—Intervention Group, CG#x2014;Control Group

**Table 6:** Prevalence of clinically meaningful psychological problems in the IG (sixth week) and CG (baseline).

Clinical Condition	Prevalence *n*/N (%)		% Difference in Prevalence between the IG and CG	χ2 (df)	*p*-Value *	Effect Size (Phi/Cramer’s V)
	IG (Six Weeks)	CG (Baseline)				
Moderate to high stress *	140/151 (92.7)	79/82 (96.3)	3.9%	1.24 (1)	0.39	0.07
Likely MDD ***	81/134 (60.4)	62/82 (75.6)	25.2%	5.23 (1)	0.03	0.16
Likely GAD **	80/131 (61.1)	59/80 (73.8)	20.8%	3.55 (1)	0.07	0.13
Thoughts of self-harm/death wish	54/134 (40.3)	49/82 (59.8)	48.4%	7.72 (1)	0.01	0.19

* Moderate or high stress defined as PSS ≥ 14; ** likely GAD defined as GAD-7 ≥ 10; *** likely MDD defined as PHQ-9 ≥ 10.

**Table 7:** Odds for subscribers in the IG to have various clinical characteristics compared to the CG when controlling for demographic variables.

Clinical Variables of Interest	*p*-Value	Odds Ratio	95% CI for OR	
			Lower	Upper
Moderate/high stress *	0.29	0.46	0.11	1.93
MDD likely ***	0.07	0.51	0.25	1.07
GAD likely **	0.03	0.44	0.21	0.92
Experienced thoughts of self-harm or death wish	0.01	0.42	0.21	0.82

* Moderate or high stress defined as PSS ≥ 14; ** likely GAD defined as GAD-7 ≥ 10; *** likely MDD defined as PHQ-9 ≥ 10. CI#x2014;Confident Interval, OR#x2014;Odd Ratio.

**Table 8:** Independent sample *t*-test comparing the mean scores for IG and CG on PSS, the GAD-7 and PHQ-9 scales and the Composite Mental Health (CMH) score.

Measure	Scores				Mean Difference (95% CI)	*p* Value	*t*-Value (df)	Effect Size (Hedge’s g)
	*n*	IG, Mean (SD)	*n*	CG, Mean (SD)				
**PSS-10 total score**	151	25.08 (6.81)	82	27.24 (6.84)	2.16 (0.32–4.01)	0.022	2.31	0.32
**PHQ-9 total score**	134	12.55 (7.15)	82	15.78 (6.94)	3.23 (1.28–5.18)	0.001	3.26	0.46
**GAD-7 total score**	131	10.89 (5.90)	80	13.45 (5.74)	2.57 (0.93–4.20)	0.002	3.10	0.44
**CMH Score**	131	48.33 (18.21)	79	56.97 (17.39)	8.65 (3.62–13.68)	0.001	3.40	0.48

CI: confidence interval; CMH: Composite Mental Health Score.

## Data Availability

The study data is available on reasonable request from the corresponding author.
